# A method for the generation of standardized qualitative dynamical systems of regulatory networks

**DOI:** 10.1186/1742-4682-3-13

**Published:** 2006-03-16

**Authors:** Luis Mendoza, Ioannis Xenarios

**Affiliations:** 1 Serono Pharmaceutical Research Institute, 14, Chemin des Aulx, 1228 Plan-les-Ouates, Geneva, Switzerland

## Abstract

**Background:**

Modeling of molecular networks is necessary to understand their dynamical properties. While a wealth of information on molecular connectivity is available, there are still relatively few data regarding the precise stoichiometry and kinetics of the biochemical reactions underlying most molecular networks. This imbalance has limited the development of dynamical models of biological networks to a small number of well-characterized systems. To overcome this problem, we wanted to develop a methodology that would systematically create dynamical models of regulatory networks where the flow of information is known but the biochemical reactions are not. There are already diverse methodologies for modeling regulatory networks, but we aimed to create a method that could be completely standardized, *i.e. *independent of the network under study, so as to use it systematically.

**Results:**

We developed a set of equations that can be used to translate the graph of any regulatory network into a continuous dynamical system. Furthermore, it is also possible to locate its stable steady states. The method is based on the construction of two dynamical systems for a given network, one discrete and one continuous. The stable steady states of the discrete system can be found analytically, so they are used to locate the stable steady states of the continuous system numerically. To provide an example of the applicability of the method, we used it to model the regulatory network controlling T helper cell differentiation.

**Conclusion:**

The proposed equations have a form that permit any regulatory network to be translated into a continuous dynamical system, and also find its steady stable states. We showed that by applying the method to the T helper regulatory network it is possible to find its known states of activation, which correspond the molecular profiles observed in the precursor and effector cell types.

## Background

The increasing use of high throughput technologies in different areas of biology has generated vast amounts of molecular data. This has, in turn, fueled the drive to incorporate such data into pathways and networks of interactions, so as to provide a context within which molecules operate. As a result, a wealth of connectivity information is available for multiple biological systems, and this has been used to understand some global properties of biological networks, including connectivity distribution [1], recurring motifs [2] and modularity [3]. Such information, while valuable, provides only a *static *snapshot of a network. For a better understanding of the functionality of a given network it is important to study its *dynamical *properties. The consideration of dynamics allows us to answer questions related to the number, nature and stability of the possible patterns of activation, the contribution of individual molecules or interactions to establishing such patterns, and the possibility of simulating the effects of loss- or gain-of-function mutations, for example.

Mathematical modeling of metabolic networks requires specification of the biochemical reactions involved. Each reaction has to incorporate the appropriate stoichiometric coefficients to account for the principle of mass conservation. This characteristic simplifies modeling, because it implies that at equilibrium every node of the metabolic network has a total mass flux of zero [4,5]. There are cases, however, where the underlying biochemical reactions are not known for large parts of a pathway, but the direction of the flow of information is known, which is the case for so-called regulatory networks (see for example [6,7]). In these cases, the directionality of signaling is sufficient for developing mathematical models of how the patterns of activation and inhibition determine the state of activation of the network (for a review, see [8]).

When cells receive external stimuli such as hormones, mechanical forces, changes in osmolarity, membrane potential etc., there is an internal response in the form of multiple intracellular signals that may be buffered or may eventually be integrated to trigger a global cellular response, such as growth, cell division, differentiation, apoptosis, secretion etc. Modeling the underlying molecular networks as dynamical systems can capture this channeling of signals into coherent and clearly identifiable stable cellular behaviors, or cellular states. Indeed, qualitative and semi-quantitative dynamical models provide valuable information about the global properties of regulatory networks. The stable steady states of a dynamical system can be interpreted as the set of all possible stable patterns of expression that can be attained within the particular biological network that is being modeled. The advantages of focusing the modeling on the stable steady states of the network are two-fold. First, it reduces the quantity of experimental data required to construct a model, *e.g. *kinetic and rate limiting step constants, because there is no need to describe the transitory response of the network under different conditions, only the final states. Second, it is easier to verify the predictions of the model experimentally, since it requires stable cellular states to be identified; that is, long-term patterns of activation and not short-lived transitory states of activation that may be difficult to determine experimentally.

**Figure 1:**
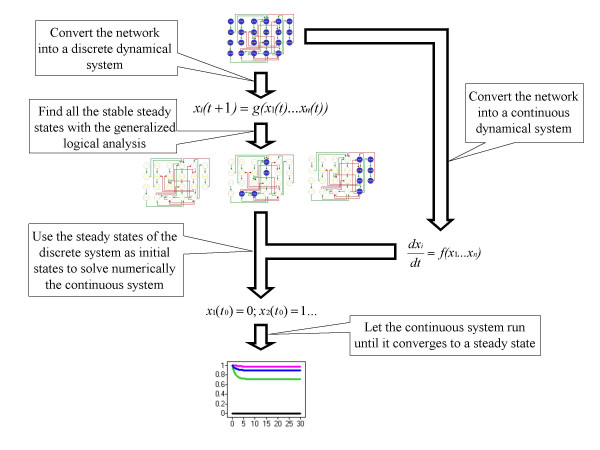
**Methodology**. Schematic representation of the method for systematically constructing a dynamical model of a regulatory network and finding its stable steady states.

In this paper we propose a method for generating qualitative models of regulatory networks in the form of continuous dynamical systems. The method also permits the stable steady states of the system to be localized. The procedure is based on the parallel construction of two dynamical systems, one discrete and one continuous, for the same network, as summarized in Figure 1. The characteristic that distinguishes our method from others used to model regulatory networks (as summarized in [8]) is that the equations used here, and the method deployed to analyze them, are completely standardized, *i.e. *they are not network-specific. This feature permits systematic application and complete automation of the whole process, thus speeding up the analysis of the dynamical properties of regulatory networks. Moreover, in contrast to methodologies for the automatic analysis of biochemical networks (as in [9]; for example), our method can be applied to networks for which there is a lack of stoichiometric information. Indeed, the method requires as sole input the information regarding the nature and directionality of the regulatory interactions. We provide an example of the applicability of our method, using it to create a dynamical model for the regulatory network that controls the differentiation of T helper (Th) cells.

## Results and discussion

**Figure 2:**
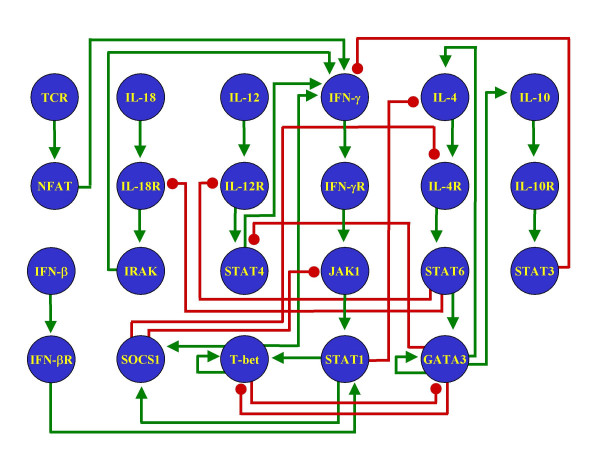
**The Th network**. The regulatory network that controls the differentiation process of T helper cells. Positive regulatory interactions are in green and negative interactions in red.

Equations 1 and 3 (see Methods) provide the means for transforming a static graph representation of a regulatory network into two versions of a dynamical system, a discrete and a continuous description, respectively. As an example, we applied these equations to the Th regulatory network, shown in Figure 2. Briefly, the vertebrate immune system contains diverse cell populations, including antigen presenting cells, natural killer cells, and B and T lymphocytes. T lymphocytes are classified as either T helper cells (Th) or T cytotoxic cells (Tc). T helper cells take part in cell- and antibody-mediated immune responses by secreting various cytokines, and they are further sub-divided into precursor Th0 cells and effector Th1 and Th2 cells, depending on the array of cytokines that they secrete [10]. The network that controls the differentiation from Th0 towards the Th1 or Th2 phenotypes is rather complex, and discrete modeling has been used to understand its dynamical properties [11,12]. In this work we used an updated version of the Th network, the molecular basis of which is included in the Methods. Also, we implement for the first time a continuous model of the Th network.

By applying Equation 1 to the network in Figure 2, we obtained Equation 2, which constitutes the discrete version of the dynamical system representing the Th network. Similarly, the continuous version of the Th network was obtained by applying Equation 3 to the network in Figure 2. In this case, however, some of the resulting equations are too large to be presented inside the main text, so we included them as the Additional file 1. Moreover, instead of just typing the equations, we decided to present them in a format that might be used directly to run simulations. The continuous dynamical system of the Th network is included as a plain text file that is able to run on the numerical computation software package GNU Octave .

The high non-linearity of Equation 3 implies that the continuous version of the dynamical model has to be studied numerically. In contrast, the discrete version can be studied analytically by using generalized logical analysis, allowing all its stable steady states to be located (see Methods). In our example, the discrete system described by Equation 2 has three stable steady states (see Table 1). Importantly, these states correspond to the molecular profiles observed in Th0, Th1 and Th2 cells. Indeed, the first stable steady state reflects the pattern of Th0 cells, which are precursor cells that do not produce any of the cytokines included in the model (IFN-β, IFN-γ, IL-10, IL-12, IL-18 and IL-4). The second steady state represents Th1 cells, which show high levels of activation for IFN-γ, IFN-γR, SOCS1 and T-bet, and with low (although not zero) levels of JAK1 and STAT1. Finally, the third steady state corresponds to the activation observed in Th2 cells, with high levels of activation for GATA3, IL-10, IL-10R, IL-4, IL-4R, STAT3 and STAT6.

**Table 1:** Stable steady states of the dynamical systems. ^a^

	**DISCRETE SYSTEM**			**CONTINUOUS SYSTEM**		
	
	Th0	Th1	Th2	Th0	Th1	Th2
**GATA3**	0	0	**1**	0	0	**1**
**IFN-β**	0	0	0	0	0	0
**IFN-βR**	0	0	0	0	0	0
**IFN-γ**	0	**1**	0	0	**0.71443**	0
**IFN-γR**	0	**1**	0	0	**0.9719**	0
**IL-10**	0	0	**1**	0	0	**1**
**IL-10R**	0	0	**1**	0	0	**1**
**IL-12**	0	0	0	0	0	0
**IL-12R**	0	0	0	0	0	0
**IL-18**	0	0	0	0	0	0
**IL-18R**	0	0	0	0	0	0
**IL-4**	0	0	**1**	0	0	**1**
**IL-4R**	0	0	**1**	0	0	**1**
**IRAK**	0	0	0	0	0	0
**JAK1**	0	0	0	0	**0.00489**	0
**NFAT**	0	0	0	0	0	0
**SOCS1**	0	**1**	0	0	**0.89479**	0
**STAT1**	0	0	0	0	**0.00051**	0
**STAT3**	0	0	**1**	0	0	**1**
**STAT4**	0	0	0	0	0	0
**STAT6**	0	0	**1**	0	0	**1**
**T-bet**	0	**1**	0	0	**0.89479**	0
**TCR**	0	0	0	0	0	0

a. Homologous non-zero values between the discrete and the continuous systems are shown in bold

Equation 3 defines a highly non-linear continuous dynamical system. In contrast with the discrete system, these continuous equations have to be studied numerically. Numerical methods for solving differential equations require the specification of an initial state, since they proceed via iterations. In our method, we propose to use the stable steady states of the discrete system as the *initial states *to solve the continuous system that results from application of equation 3 to a given network. We used a standard numerical simulation method to solve the continuous version of the Th model (see Methods). Starting alternatively from each of the three stable steady states found in the discrete model, *i.e. *the Th0, Th1 and Th2 states, the continuous system was solved numerically until it converged. The continuous system converged to values that could be compared directly with the stable steady states of the discrete system (Table 1). Note that the Th0 and Th2 stable steady states fall in exactly the same position for both the discrete and the continuous dynamical systems, and in close proximity for the Th1 state. This finding highlights the similarity in *qualitative *behavior of the two models constructed using equations 1 and 3, despite their different mathematical frameworks.

Despite the qualitative similarity between the discrete and continuous systems, there is no guarantee that the continuous dynamical system has only three stable steady states; there might be others without a counterpart in the discrete system. To address this possibility, we carried out a statistical study by finding the stable steady states reached by the continuous system starting from a large number of initial states. The continuous system was run 50,000 times, each time with the nodes in a random initial state within the closed interval between 0 and 1. In all cases, the system converged to one of only three different regions (Table 2), corresponding to the above-mentioned Th0, Th1 and Th2 states. These results still do not eliminate the possibility that other stable steady states exist in the continuous system. Nevertheless, they show that if such additional stable steady states exist, their basin of attractions is relatively small or restricted to a small region of the state space.

**Table 2:** Regions of the state space reached by the continuous version of the Th model, as revealed by a large number of simulations starting from a random initial state. ^a^

	**Th0**		**Th1**		**Th2**	
	
	**Avrg.**	**Std. Dev.**	**Avrg.**	**Std. Dev.**	**Avrg.**	**Std. Dev.**
**GATA3**	0.00003	0.00008	0.00000	0.00000	**0.99997**	0.00007
**IFN-β**	0.00000	0.00000	0.00000	0.00000	0.00000	0.00000
**IFN-βR**	0.00000	0.00001	0.00000	0.00001	0.00000	0.00001
**IFN-γ**	0.00005	0.00013	**0.71438**	0.00059	0.00000	0.00001
**IFN-γR**	0.00004	0.00011	**0.97169**	0.00040	0.00001	0.00004
**IL-10**	0.00003	0.00007	0.00000	0.00001	**0.99999**	0.00004
**IL-10R**	0.00005	0.00010	0.00000	0.00001	**0.99999**	0.00002
**IL-12**	0.00000	0.00001	0.00000	0.00000	0.00000	0.00001
**IL-12R**	0.00000	0.00002	0.00000	0.00001	0.00000	0.00001
**IL-18**	0.00000	0.00001	0.00000	0.00000	0.00000	0.00001
**IL-18R**	0.00000	0.00002	0.00000	0.00001	0.00000	0.00001
**IL-4**	0.00002	0.00006	0.00000	0.00001	**0.99995**	0.00011
**IL-4R**	0.00002	0.00004	0.00000	0.00001	**0.99990**	0.00022
**IRAK**	0.00001	0.00005	0.00000	0.00003	0.00001	0.00004
**JAK1**	0.00002	0.00008	0.00487	0.00005	0.00001	0.00005
**NFAT**	0.00001	0.00003	0.00000	0.00002	0.00001	0.00003
**SOCS1**	0.00009	0.00022	**0.89486**	0.00037	0.00002	0.00006
**STAT1**	0.00001	0.00005	0.00051	0.00003	0.00002	0.00005
**STAT3**	0.00012	0.00023	0.00001	0.00002	**1.00000**	0.00002
**STAT4**	0.00001	0.00003	0.00000	0.00003	0.00000	0.00001
**STAT6**	0.00001	0.00004	0.00000	0.00002	**0.99990**	0.00023
**T-bet**	0.00007	0.00018	**0.89485**	0.00036	0.00000	0.00000
**TCR**	0.00000	0.00001	0.00000	0.00000	0.00000	0.00001

a. Only three regions of the activation space were found in the continuous Th model after running it from 50,000 different random initial states. The average and standard deviations of all the results are shown. All variables had a random initial state in the closed interval [0,1]. From the 50,000 simulations, 8195 (16.39%) converged to the Th0 state, 25575 (51.15%) to the Th1 state, and 16230 (32.46%) to the Th2 state. Bold numbers as in Table 1.

**Figure 3:**
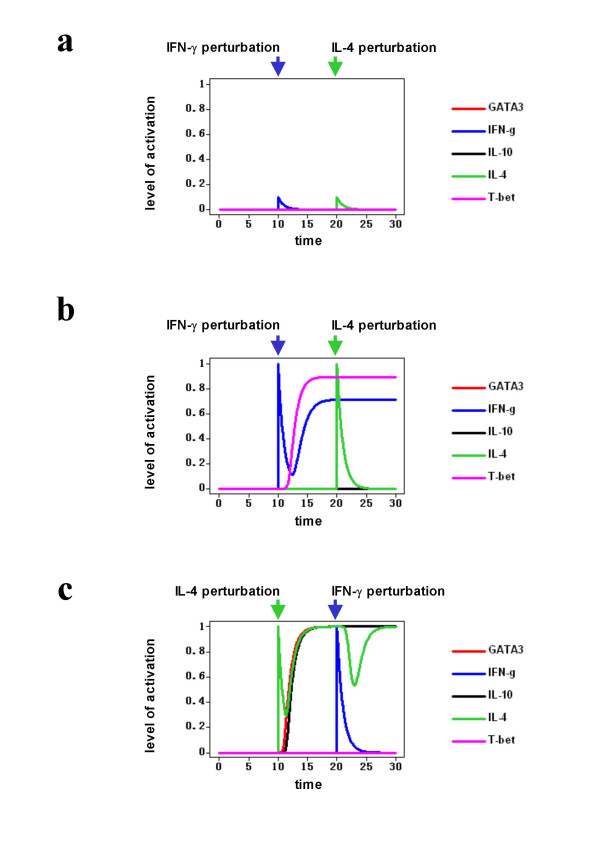
**Stability of the steady states of the continuous model of the Th network**. **a**. The Th0 state is stable under small perturbations. **b**. A large perturbation on IFN-γ is able to move the system from the Th0 to the Th1 steady state. This latter state is stable to perturbations. **c**. A large perturbation of IL-4 moves the system from the Th0 state to the Th2 state, which is stable. For clarity, only the responses of key cytokines and transcription factors are plotted. The time is represented in arbitrary units.

The three steady states of the continuous system are stable, since they can resist small perturbations, which create transitory responses that eventually disappear. Figure 3a shows a simulation where the system starts in its Th0 state and is then perturbed by sudden changes in the values of IFN-γ and IL-4 consecutively. Note that the system is capable of absorbing the perturbations, returning to the original Th0 state. If a perturbation is large enough, however, it may move the system from one stable steady state to another. If the system is in the Th0 state and IFN-γ is transiently changed to it highest possible value, namely 1, the whole system reacts and moves to its Th1 state (Figure 3b). A large second perturbation by IL-4, now occurring when the system is in its Th1 state, does not push the system into another stable steady state, showing the stability of the Th1 state. Conversely, if the large perturbation of IL-4 occurs when the system is in the Th0 state, it moves the system towards the Th2 state (Figure 3c). In this case, a second perturbation, now in IFN-γ, creates a transitory response that is not strong enough to move the system away from the Th2 state, showing the stability of this steady state. These changes from one stable steady state to another reflect the biological capacities of IFN-γ and IL-4 to act as key signals driving differentiation from Th0 towards Th1 and Th2 cells, respectively[10]. Furthermore, note that the Th1 and Th2 steady states are more resistant to large perturbations than the Th0 state, a characteristic that represents the stability of Th1 and Th2 cells under different experimental conditions.

The whole process resulted in the creation of a model with qualitative characteristics fully comparable to those found in the experimental Th system. Notably, the model used default values for all parameters. Indeed, the continuous dynamical system of the Th network has a total of 58 parameters, all of which were set to the default value of 1, and one parameter (the gain of the sigmoids) with a default value of 10. This set of default values sufficed to capture the correct qualitative behavior of the biological system, namely, the existence of three stable steady states that represent Th0, Th1 and Th2 cells. Readers can run simulations on the model by using the equations provided in the "Th_continuous_model.octave.txt" file. The file was written to allow easy modification of the initial states for the simulations, as well as the values of all parameters.

### Analysis of previously published regulatory networks related to Th cell differentiation

**Figure 4:**
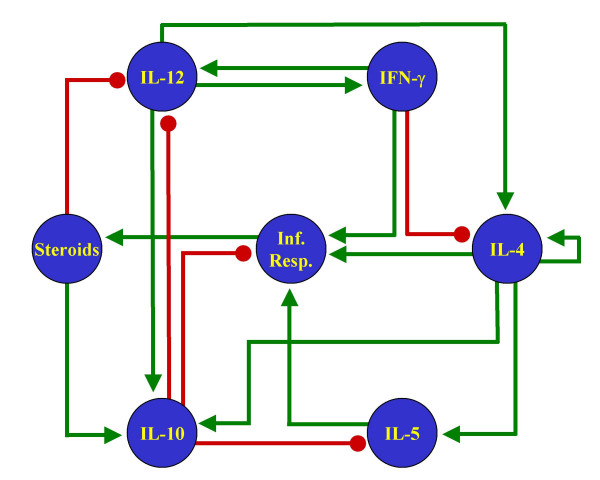
**Alternative Th network**. T helper pathway published in [69], reinterpreted as a signaling network.

We wanted to compare the results from our method (Figure 1) as applied to our proposed network (Figure 2) with some other similar networks. The objective of this comparison is to show that our method imposes no restrictions on the number of steady states in the models. Therefore, if the procedure is applied to wrongly reconstructed networks, the results will not reflect the general characteristics of the biological system. While there have been multiple attempts to reconstruct the signaling pathways behind the process of Th cell differentiation, they have all been carried out to describe the molecular components of the process, but not to study the dynamical behavior of the network. As a result, most of the schematic representations of these pathways are not presented as regulatory networks, but as collections of molecules with different degrees of ambiguity to describe their regulatory interactions. To circumvent this problem, we chose four pathways with low numbers of regulatory ambiguities and translated them as signaling networks (Figures 4 through 7).

**Figure 5:**
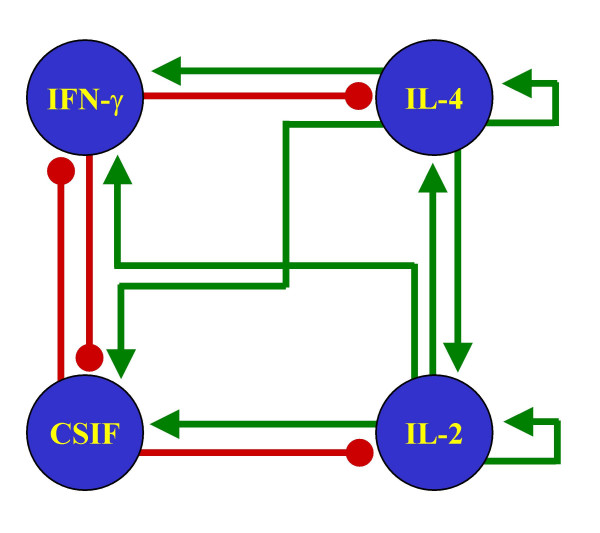
**Alternative Th network**. T helper pathway published in [70], reinterpreted as a signaling network.

**Figure 6:**
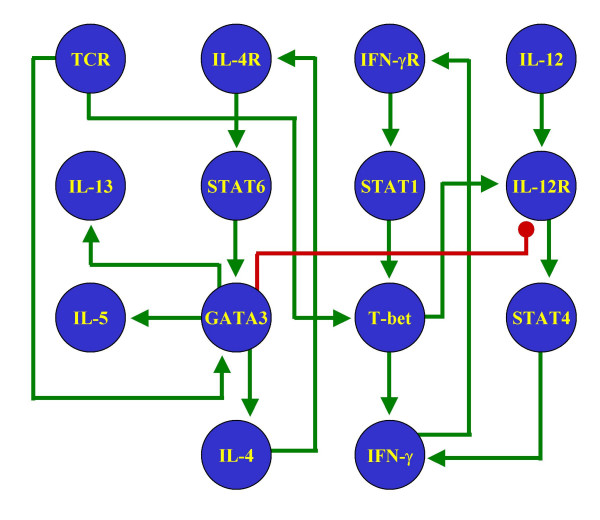
**Alternative Th network**. T helper pathway published in [43], reinterpreted as a signaling network.

**Figure 7:**
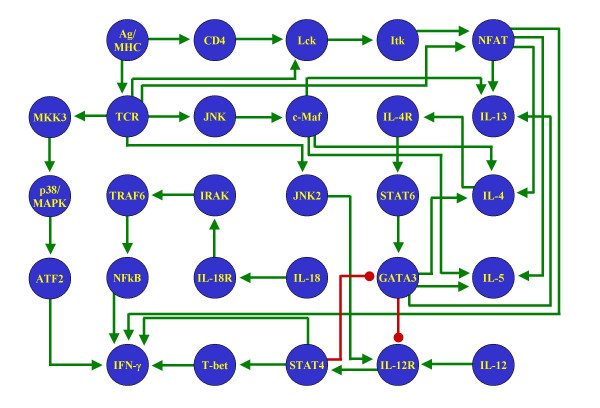
**Alternative Th network**. T helper pathway published in [71], reinterpreted as a signaling network.

The methodology introduced in this paper was applied to the four reinterpreted networks for Th cell differentiation. The stable steady states of the resulting discrete and continuous models are presented in Tables 3 through 6. Notice that none of these four alternative networks could generate the three stable steady states representing Th0, Th1 and Th2 cells. Two networks reached only two stable steady states, while two others reached more than three. Notably, all these four networks included one state representing the Th0 state, and at least one representing the Th2 state. The absence of a Th1 state in two of the networks might reflect the lack of a full characterization of the IFN-γ signaling pathway at the time of writing the corresponding papers.

**Table 3:** Stable steady states of the signaling network in Figure 4

	Discrete state 1	Discrete state 2	Continuous state 1	Continuous state 2
**IFN-γ**	0	0	0	0
**IL-10**	0	1	0	0.78995
**IL-12**	0	0	0	0
**IL-4**	0	1	0	0.89469
**IL-5**	0	0	0	0.01343
**Inf. Resp.**	0	0	0	0.00737
**Steroids**	0	0	0	0.00105

**Table 4:** Stable steady states of the signaling network in Figure 5

	Discrete state 1	Discrete state 2	Discrete state 3	Discrete state 4	Discrete state 5	Discrete state 6	Discrete state 7
**CSIF**	0	0	1	0	0.5	0.5	0
**IFN-γ**	0	1	0	0.5	0	0	0. 5
**IL-2**	0	1	0	0.5	0.5	0.5	0
**IL-4**	0	0	1	0.5	0	0.5	0.5

	Continuous state 1	Continuous state 2	Continuous state 3	Continuous state 4	Continuous state 5	Continuous state 6	Continuous state 7

**CSIF**	0	0.0034416	0.8888881	0.0034999	4.9132E-5	0.8881746	4.3001E-5
**IFN-γ**	0	0.8888881	0.0034416	0.8881746	4.300E-5	0.0034999	4.9132E-5
**IL-2**	0	0.8888881	0.0034416	0.8881746	4.3154E-5	0.0035227	4.8979E-5
**IL-4**	0	0.0034416	0.8888881	0.0035227	4.8979E-5	0.8881746	4.3154E-5

**Table 5:** Stable steady states of the signaling network in Figure 6

	Discrete state 1	Discrete state 2	Discrete state 3	Discrete state 4	Continuous state 1	Continuous state 2	Continuous state 3	Continuous state 4
**GATA3**	0	0	11	1	0	0	0.93037	0.93037
**IFN-γ**	0	1	0	1	0	0.99914	0	0.90967
**IFN-γR**	0	1	0	1	0	0.99997	0	0.99617
**IL-12**	0	0	0	0	0	0	0	0
**IL-12R**	0	1	0	0	0	0.9096	0	0.00193
**IL-13**	0	0	1	1	0	0	0.99719	0.99719
**IL-4**	0	0	1	1	0	0	0.99719	0.99719
**IL-4R**	0	0	1	1	0	0	0.99991	0.99991
**IL-5**	0	0	1	1	0	0	0.99719	0.99719
**STAT1**	0	1	0	1	0	1	0	0.99988
**STAT4**	0	1	0	0	0	0.99617	0	2.4E-4
**STAT6**	0	0	1	1	0	0	1	1
**T-bet**	0	1	0	1	0	0.93037	0	0.93034
**TCR**	0	0	0	0	0	0	0	0

**Table 6:** Stable steady states of the signaling network in Figure 7

	Discrete state 1	Discrete state 2	Continuous state 1	Continuous state 2
**Ag/MHC**	0	0	0	0
**ATF2**	0	0	0	0
**c-Maf**	0	0	0	0
**CD4**	0	0	0	0
**GATA3**	0	1	0	0.99999
**IFN-γ**	0	0	0	0
**IL-12**	0	0	0	0
**IL-12R**	0	0	0	0
**IL-13**	0	1	0	0.8468
**IL-18**	0	0	0	0
**IL-18R**	0	0	0	0
**IL-4**	0	1	0	0.8468
**IL-4R**	0	1	0	0.99176
**IL-5**	0	1	0	0.8469
**IRAK**	0	0	0	0
**Itk**	0	0	0	0
**JNK**	0	0	0	0
**JNK2**	0	0	0	0
**Lck**	0	0	0	0
**MKK3**	0	0	0	0
**NFAT**	0	0	0	0
**NFkB**	0	0	0	0
**p38/MAPK**	0	0	0	0
**STAT4**	0	0	0	0
**STAT6**	0	1	0	0.99975
**T-bet**	0	0	0	0
**TCR**	0	0	0	0
**TRAF6**	0	0	0	0

It is important to note that the failure of these four alternative networks to capture the three states representing Th cells is not attributable to the use of very simplistic and/or outdated data. Indeed, the network in Figure 6 comes from a relatively recent review, while that in Figure 7 is rather complex and contains five more nodes than our own proposed network (Figure 2). All this stresses the importance of using a correctly reconstructed network to develop dynamical models, either with our approach or any other.

## Conclusion

There is a great deal of interest in the reconstruction and analysis of regulatory networks. Unfortunately, kinetic information about the elements that constitute a network or pathway is not easily gathered, and hence the analysis of its dynamical properties (via simulation packages such as [13]) is severely restricted to a small set of well-characterized systems. Moreover, the translation from a static to a dynamical representation normally requires the use of a network-specific set of equations to represent the expression or concentration of every molecule in the system.

We herein propose a method for generating a system of ordinary differential equations to construct a model of a regulatory network. Since the equations can be unambiguously applied to any signaling or regulatory network, the construction and analysis of the model can be carried out systematically. Moreover, the process of finding the stable steady states is based on the application of an analytical method (generalized logical analysis [14,15] on a discrete version of the model), followed by a numerical method (on the continuous version) starting from specific initial states (the results obtained from the logical analysis). This characteristic allows a fully automated implementation of our methodology for modeling. In order to construct the equations of the continuous dynamical system with the exclusive use of the topological information from the network, the equations have to incorporate a set of default values for all the parameters. Therefore, the resulting model is not optimized in any sense. However, the advantage of using Equation 3 is that the user can later modify the parameters so as to refine the performance of the model, approximating it to the known behavior of the biological system under study. In this way, the user has a range of possibilities, from a purely qualitative model to one that is highly quantitative.

There are studies that compare the dynamical behavior of discrete and continuous dynamical systems. Hence, it is known that while the steady state of a Boolean model will correspond qualitatively to an analogous steady state in a continuous approach, the reverse is not necessarily true. Moreover, periodic solutions in one representation may be absent in the other [16]. This discrepancy between the discrete and continuous models is more evident for steady states where at least one of the nodes has an activation state precisely at, or near, its threshold of activation. Because of this characteristic, discrete and continuous models for a given regulatory network differ in the total number of steady states [17]. For this reason, our method focuses on the study of only one type of steady state; namely, the regular stationary points [18]. These points do not have variables near an activation threshold, and they are always stable steady states. Moreover, it has been shown that this type of stable steady state can be found in discrete models, and then used to locate their analogous states in continuous models of a given genetic regulatory network [19].

It is beyond the scope of this paper to present a detailed mathematical analysis of the dynamical system described by Equation 3. Instead, we present a framework that can help to speed up the analysis of the qualitative behavior of signaling networks. Under this perspective, the usefulness of our method will ultimately be determined through building and analyzing concrete models. To show the capabilities of our proposed methodology, we applied it to analysis of the regulatory network that controls differentiation in T helper cells. This biological system was well suited to evaluating our methodology because the network contains several known components, and it has three alternative stable patterns of activation. Moreover, it is of great interest to understand the behavior of this network, given the role of T helper cell subsets in immunity and pathology [20]. Our method applied to the Th network generated a model with the same qualitative behavior as the biological system. Specifically, the model has three stable states of activation, which can be interpreted as the states of activation found in Th0, Th1 and Th2 cells. In addition, the system is capable of being moved from the Th0 state to either the Th1 or Th2 states, given a sufficiently large IFN-γ or IL-4 signal, respectively. This characteristic reflects the known qualitative properties of IFN-γ and IL-4 as key cytokines that control the fate of T helper cell differentiation.

Regarding the numerical values returned by the model, it is not possible yet to evaluate their accuracy, given that (to our knowledge) no quantitative experimental data are available for this biological system. The resulting model, then, should be considered as a *qualitative *representation of the system. However, representing the nodes in the network as normalized continuous variables will eventually permit an easy comparison with quantitative experimental data whenever they become available. Towards this end, the equations in our methodology define a sigmoid function, with values ranging from 0 to 1, regardless of the values of assigned to the parameters in the equations. This characteristic has been used before to represent and model the response of signaling pathways [21,22]. It is important to note, however, that the modification of the parameters allow the model to be fitted against experimental data.

One benefit of a mathematical model of a particular biological network is the possibility of predicting the behavior of complex experimental setups. Therefore, it is important to be aware of its limitations beforehand, to avoid generating experimental data that cannot be handled by the model. The method we present in this paper has been developed to obtain the number and relative position of the stable steady states of a regulatory network. Equations 1 and 3 include a number of parameters that allow the response of the model to be fine-tuned, but the equations were not designed to describe the transitory responses of molecules with great detail. Therefore, failure to predict a stable steady state with high numerical accuracy should not be interpreted as a failure of the approach presented here. By contrast, failure to describe and/or predict the number and approximate location of stable steady states under a wide range of values for the parameters would call the validity of the reconstruction of a particular network into question. Here, however, it is essential to establish the validity of the network used as input. Indeed, we applied our method to four alternative forms of the network that regulates Th cell differentiation. The alternative networks (Figures 4 through 7) were taken from previously published attempts to discover the molecular basis of this differentiation process. Originally, such networks were not developed with the idea of studying dynamical properties. It is not surprising, then, that these networks do not reflect the existence of three stable steady states, representing the molecular states of Th0, Th1 and Th2 cells, respectively. In these cases, the failure to find the correct stable steady states is not a problem in the modeling methodology, but a problem in the inference of the regulatory network.

In conclusion, we have shown that the creation of a dynamical model of a regulatory network can be considerably simplified with the aid of a standardized set of equations, where the feature that distinguishes one molecule from another is the number of regulatory inputs. Such standardization permits a continuous dynamical system to be systematically and analytically constructed together with a basic analysis of its global properties, based exclusively on the information provided by the connectivity of the network. While the use of a standardized set of functions to model a network may severely restrict the capability to fit specific datasets, we believe that the loss in flexibility is balanced by the possibility of rapidly developing models and gaining knowledge of the dynamical behavior of a network, especially in those cases where few kinetic data are available. Thus, we provide a method for incorporating the dynamical perspective in the analysis of regulatory networks, using the topological information of a network, without the need to collect extensive time-series or kinetic data.

## Methods

### Molecular basis of the Th network topology

The following paragraphs detail the evidence used to infer the topology of the Th regulatory network, updating the data summarized in [11]. Th1 cells are producers of IFN-γ [10,23], which acts on its target cells by binding to a cell-membrane receptor [24-26] to start a signaling cascade, which involves JAK1 and STAT-1 [27-29]. STAT-1 can be activated by a number of ligands besides IFN-γ, but importantly, it cannot be activated by IL-4 [30], which is a major Th2 signal. In contrast, STAT-1 plays a role in modulating IL-4, being an intermediate in the negative regulation of IFN-γ exerted on IL-4 expression [31]. Different signals converge in STAT-1, among them that of IFN-β/IFN-βR [32]. The IFN-γ signaling continues downstream to activate SOCS-1 in a STAT-1-dependent pathway [33,34]. SOCS-1, in turn, influences both the IFN-γ and IL-4 pathways. On the one hand, SOCS-1 is a negative regulator of IFN-γ signaling, blocking the interaction of IFN-γR and STAT-1 [35] due to direct inhibition of JAK1 [29,36]. On the other hand, SOCS-1 blocks the IL-4R/STAT-6 pathway [37]. SOCS-1 is, therefore, a key element for the inhibition from the IFN-γ to the IL-4 pathway. Th1 cells express high levels of SOCS-1 mRNA, while it is barely detectable in Th0 and Th2 cells [38]. Finally, another key molecule is T-bet, which is a transcription factor detected in Th1 but not Th0 or Th2 cells. T-bet expression is upregulated by IFN-γ in a STAT-1-dependent mechanism [39]. Importantly, T-bet is an inhibitor of GATA-3 [40], an activator of IFN-γ [40] and activator of T-bet itself [41,42].

Th2 cells express IL-4, which is the major known determinant of the Th2 phenotype itself [43]. IL-4 binds to its receptor, IL-4R, which is preferentially expressed in Th2 cells [23,44]. The IL-4R signaling is transduced by STAT-6, which in turn activates GATA-3 [10]. GATA-3, in turn, is capable of inducing IL-4 [45], thus establishing a feedback loop. The influence from the IL-4 pathway on the IFN-γ pathway seems to be mediated by GATA-3 via STAT-4 [46]. Like T-bet, GATA-3 also presents a self-activation loop [47-49].

IL-12 and IL-18 are two molecules that affect the IFN-γ pathway. IL-12 is a cytokine produced by monocytes and dendritic cells and promotes the development of Th1 cells [50]. The IL-12 receptor is present in its functional form in Th0 and Th1 but not Th2 cells [51]. IL-12R signaling is mediated by STAT-4 [52], which is able to activate IFN-γ [41,46,53]. The IL-12 signaling pathway can be blocked by IL-4 by the STAT-6 dependent down-regulation of one subunit of IL-12R [54]. IL-18 is a cytokine produced by many cell types and promotes IFN-γ production in Th cells [55]. It acts upon binding to its receptor, IL-18R, which acts through IRAK [56]. IL-12 and IL-18 act synergistically to increase IFN-γ production, but using different pathways [57,58]. Finally, IL-4 is able to block IL-18 signaling in a STAT-6 dependent manner [59].

IL-10 is a cytokine actively produced by Th2 cells, and it inhibits cytokine production by Th1 cells. As with the other cytokines mentioned above, IL-10 acts upon binding to a cell surface receptor, IL-10R, which in turn activates the STAT signaling system [60]. In particular, it has been shown that the functioning of IL-10 signaling is dependent upon the presence of STAT-3 [61]. As for the signals affecting IL-10 expression, it has been shown that IL-4 enhances IL-10 gene expression in Th2 but not Th1 cells [62]. This requirement implies that the intracellular signaling from IL-4 to IL-10 should pass through a Th2 specific molecule, which from the molecules considered here can only be GATA-3. Finally, IL-10 has been shown to be a very powerful inhibitor of IFN-γ production [60,63].

Cytokine gene expression in T cells is induced by the activation of the T cell receptor (TCR) by ligand binding. Different signaling pathways are activated by the TCR [64]. Among these is the pathway including the NFAT family of transcription factors, which are implicated in the T cell activation-dependent regulation of numerous cytokines. A constitutively active form of one of the NFAT proteins, specifically NFATc1, increases the expression of IFN-γ [65]. Importantly, the same experimental procedure does not affect the expression of IL-4. All this indicates that the NFAT family members play a central role in the TCR-induced expression of cytokines during Th cell differentiation, especially in the Th1 pathway.

### The discrete dynamical system

The discrete system represents the network as a series of interconnected elements that have only two possible states of activation, 0 (or inactive) and 1 (or active). Given this property, the network is completely described by the following set of Boolean equations:

Equation 1.



A node *x *in the network can have only one of three possible forms depending on whether it has activator and inhibitor input nodes, or only activators, or only inhibitors. In the first case, *i.e. *form *§ *in Eqn.1, the Boolean function can be read as: *x *will be active in the next time step if at this time any of its activators and none of its inhibitors are acting upon it. Similarly, form *§§ *can be translated as: *x *will be active if any of its activators is acting upon it. And finally, form *§§§ *reads as: *x *will be active if none of its inhibitors are acting upon it. Note than in all cases inhibitors are strong enough to change the state of a node from 1 to 0, while activators are strong enough to change the state of a node from 0 to 1 if no inhibitor is acting on the node of reference. The three alternative forms of representing a node in Equation 1 imply two possible default states of activation, *i.e. *the state of a node when there are neither activators nor inhibitors acting upon it. If the connectivity of the node includes either only positive inputs, or both positive and negative inputs, then the node has an inactive state by default. Alternatively, if the connectivity of a node has only negative inputs, then the node has an active state by default.

The Th network (Figure 2) can be converted into a discrete dynamical system using Equation 1. The resulting system of equations is as follows:

Equation 2.

*GATA*3(*t *+ 1) = (*GATA*3(*t*) ∨ *STAT*6(*t*)) ∧ ¬(*T *- *bet*(*t*))

*IFN *- *βR*(*t *+ 1) = *IFN *- *β*(*t*)

*IFN *- *γ*(*t *+ 1) = (*IRAK*(*t*) ∨ *NFAT*(*t*) ∨ *STAT *- 4(*t*) ∨ *T *- *bet*(*t*)) ∧ ¬(*STAT*3(*t*))

*IFN *- *γR*(*t *+ 1) = *IFN *- *γ*(*t*)

*IL *- 10(*t *+ 1) = *GATA*3(*t*)

*IL *- 10*R*(*t *+ 1) = *IL *- 10(*t*)

*IL *- 12*R*(*t *+ 1) = *IL *- 12(*t*)

*IL *- 18*R*(*t *+ 1) = *IL *- 18(*t*) ∧ ¬(*STAT6*(*t*))

*IL *- 4(*t *+ 1) = *GATA*3(*t*) ∧ ¬(*STAT*1(*t*))

*IL *- 4*R*(*t *+ 1) = *IL *- 4(*t*) ∧ ¬(*SOCS*1(*t*))

*IRAK*(*t *+ 1) = *IL *- 18*R*(*t*)

*JAK*1(*t *+ 1) = *IFN *- *γR*(*t*) ∧ ¬(*SOCS*1(*t*))

*NFAT*(*t *+ 1) = *TCR*(*t*)

*SOCS*1(*t *+ 1) = *STAT*1(*t*) ∨ *T *- *bet*(*t*)

*STAT*1(*t *+ 1) = *IFN *- *βR*(*t*) ∨ *JAK*1(*t*)

*STAT*3(*t *+ 1) = *IL *- 10*R*(*t*)

*STAT*4(*t *+ 1) = *IL *- 12*R*(*t*) ∧ ¬(*GATA*3(*t*))

*STAT*6(*t *+ 1) = *IL *- 4*R*(*t*)

*T *- *bet*(*t *+ 1) = (*STAT*1(*t*) ∨ *T *- *bet*(*t*)) ∧ ¬(*GATA*3(*t*))

Notice that there are only 19 equations out of a total of 23 elements in the Th network. The reason is that four elements, namely IFN-β, IL-12, IL-18 and TCR, do not have inputs. These four elements are thus treated as constants, since there are no interactions that regulate their behavior. Throughout the text, these four elements are considered as having a value of 0.

### Stable steady states of the discrete system

The discrete dynamical system defined by Equation 2 can be solved in different ways to find its attractors, depending on how to update the vector state **X**(*t*) to its successor, **X**(*t+1*). By far the easiest method for solving the equations is the synchronous approach (as in [66,67]). This method, however, can generate spurious results (see [14]). Hence, we use generalized logical analysis to find all the steady states of the system [15]. Generalized logical analysis allows us to find all the steady states of a discrete dynamical system by evaluating the functionality of the feedback loops, also known as circuits, in the system. In this case, the Th network (Figure 2) contains a total of 27 circuits (Table 7), 24 positive and 3 negative. Depending on the set of parameters used, positive feedback loops can generate multistationarity, while negative feedback loops can generate damped or sustained oscillations. Generalized logical analysis is a well-established method and the reader may find in-depth explanations elsewhere [14,15,18].

**Table 7:** Circuits of the Th network ^a^

1	IFNγ→IFNγR→JAK1→STAT1¬IL4→IL4R→STAT6¬IL18R→IRAK→
2	IFNγ→IFNγR→JAK1→STAT1¬IL4→IL4R→STAT6¬IL12R→STAT4→
3	IFNγ→IFNγR→JAK1→STAT1¬IL4→IL4R→STAT6→GATA3→IL10→IL10R→STAT3¬
4	IFNγ→IFNγR→JAK1→STAT1¬IL4→IL4R→STAT6→GATA3¬STAT4→
5	IFNγ→IFNγR→JAK1→STAT1¬IL4→IL4R→STAT6→GATA3¬Tbet→
6	IFNγ→IFNγR→JAK1→STAT1→SOCS1¬IL4R→STAT6¬IL18R→IRAK→
7	IFNγ→IFNγR→JAK1→STAT1→SOCS1¬IL4R→STAT6¬IL12R→STAT4→
8	IFNγ→IFNγR→JAK1→STAT1→SOCS1¬IL4R→STAT6→GATA3→IL10→IL10R→STAT3¬
9	IFNγ→IFNγR→JAK1→STAT1→SOCS1¬IL4R→STAT6→GATA3¬STAT4→
10	IFNγ→IFNγR→JAK1→STAT1→SOCS1¬IL4R→STAT6→GATA3¬Tbet→
11	IFNγ→IFNγR→JAK1→STAT1→Tbet→
12	IFNγ→IFNγR→JAK1→STAT1→Tbet→SOCS1¬IL4R→STAT6¬IL18R→IRAK→
13	IFNγ→IFNγR→JAK1→STAT1→Tbet→SOCS1¬IL4R→STAT6¬IL12R→STAT4→
14	IFNγ→IFNγR→JAK1→STAT1→Tbet→SOCS1¬IL4R→STAT6→GATA3→IL10→IL10R→STAT3¬
15	IFNγ→IFNγR→JAK1→STAT1→Tbet→SOCS1¬IL4R→STAT6→GATA3¬STAT4→
16	IFNγ→IFNγR→JAK1→STAT1→Tbet¬GATA3→IL4→IL4R→STAT6¬IL18R→IRAK→
17	IFNγ→IFNγR→JAK1→STAT1→Tbet¬GATA3→IL4→IL4R→STAT6¬IL12R→STAT4→
18	IFNγ→IFNγR→JAK1→STAT1→Tbet¬GATA3→IL10→IL10R→STAT3¬
19	IFNγ→IFNγR→JAK1→STAT1→Tbet¬GATA3¬STAT4→
20	IL4→IL4R→STAT6→GATA3→
21	IL4R→STAT6→GATA3¬ Tbet→SOCS1¬
22	Tbet→
23	Tbet¬GATA3¬
24	GATA3→
25	IL4→IL4R→STAT6→GATA3¬Tbet→SOCS1¬JAK1→STAT1¬
26	JAK1→STAT1→SOCS1¬
27	JAK1→STAT1→Tbet→ SOCS1¬

a. If the circuit has zero or an even number of negative interactions, it is considered positive; otherwise the circuit is negative. Circuits 1–24 are positive, and circuits 25–27 are negative.

### The continuous dynamical system

To describe the network as a continuous dynamical system, we use the following set of ordinary differential equations:

Equation 3.



**Figure 8:**
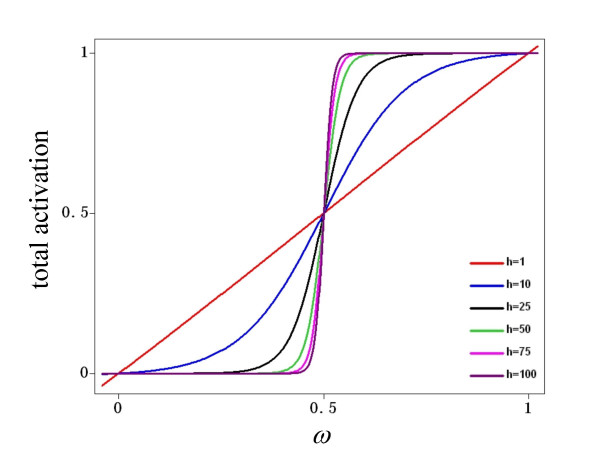
**Activation of a node as a function of its total input, *ω***. Equation 3 ensures that the activation of a node has the form of a sigmoid, bounded in the interval [0,1] regardless of the values of *h*.

The right-hand side of the differential equation comprises two parts: an activation function and a term for decay. Activation is a sigmoid function of *ω*, which represents the total input to the node. The equation of the sigmoid was chosen so as to pass through the two points (0,0) and (1,1), regardless of the value of its gain, *h*; see Figure 8. The bounding of a node *x *to the closed interval [0,1] implies that its level of activation should be interpreted as a *normalized*, not an absolute, value. This characteristic permits direct comparison between the discrete and the continuous dynamical systems, since in both formalisms the minimum and maximum levels of activation are 0 and 1. Subsequently, the second part of the equation is a decay term, which for simplicity is directly proportional to the level of activation of the node.

**Figure 9:**
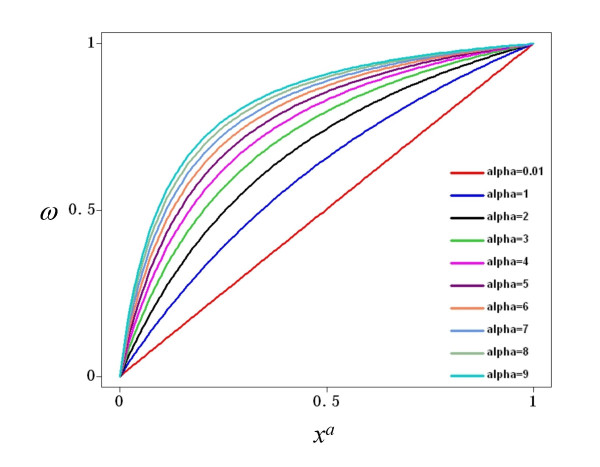
**Total input to a node, *ω*, as a function of one positive input, *x*^*a*^**. The value of *ω *is a bounded function in the interval [0,1] regardless of the interaction weight of the positive input, *α*.

**Figure 10:**
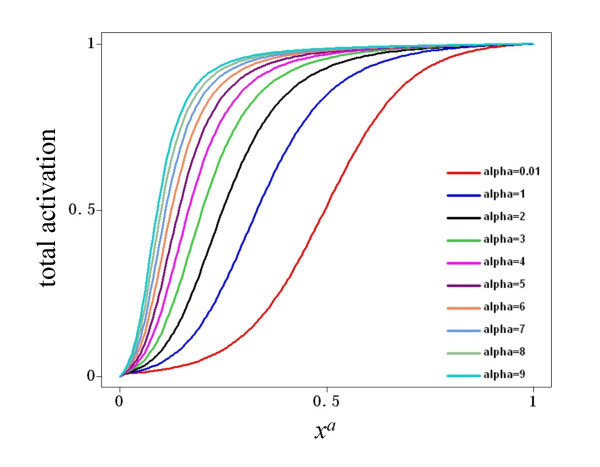
**Activation of a node as a function of one positive input**. The activation of a node in response to one positive input, plotted for various possible interaction weights.

**Figure 11:**
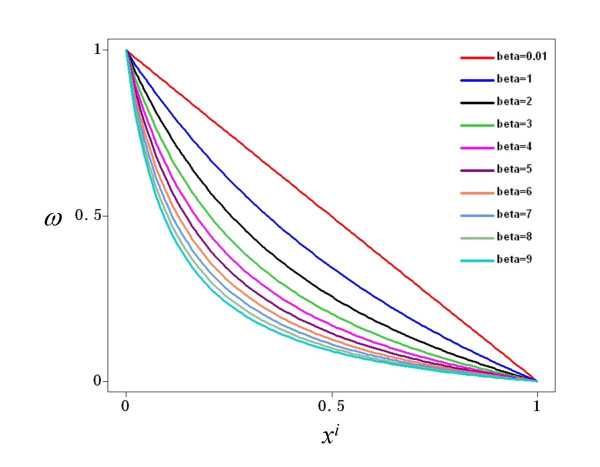
**Total input to a node, *ω*, as a function of one negative input, *x*^*i*^**. The value of *ω *is a bounded function in the interval [0,1] regardless of the interaction weight of the negative input, *β*.

**Figure 12:**
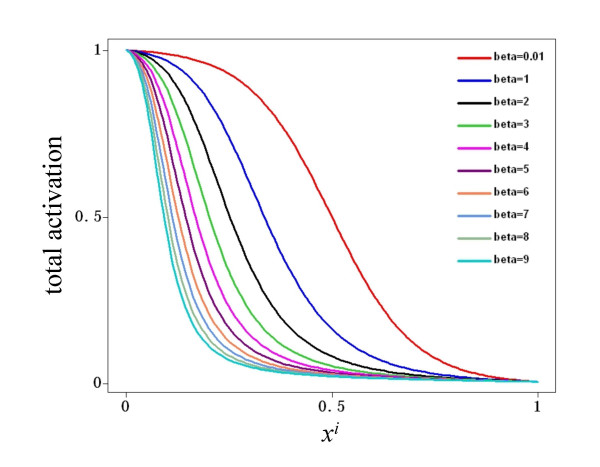
**Activation of a node as a function of one negative input**. The activation of a node in response to one negative input, plotted for various possible interaction weights.

**Figure 13:**
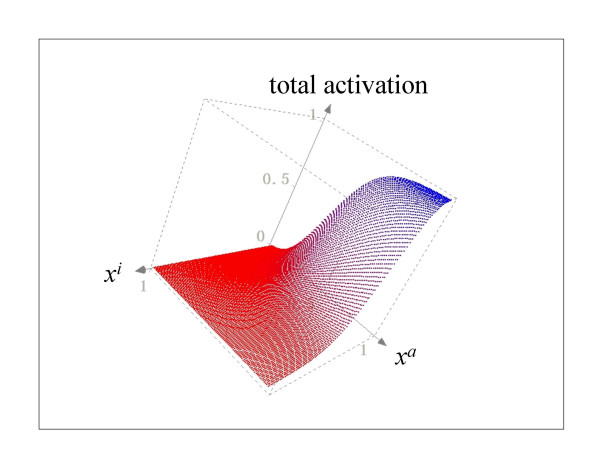
**Activation of a node as a function two inputs, one positive and one negative**. The strength of the interactions are equal for the activation and the inhibition, *α *= *β *= 1.

The total input to a node, represented by *ω*, is a combination of the multiple activatory and inhibitory interactions acting upon the node of reference. In the general case, different nodes have different connectivities; hence it is necessary to write a function *ω *so that it can describe different combinations of activatory and inhibitory inputs. For this reason, *ω *has three possible forms in Equation 3. If a node *x*_*i *_is regulated by both activators and inhibitors, then the first form, *§*, is used. However, if is regulated exclusively by activators, form *§§ *is used instead. Finally, the form *§§§ *is used if *x*_*i *_has only negative regulators. In all cases, the total input is a combination of weighted activators and/or inhibitors, where the weights are represented by the *α *and *β *parameters for the activators and inhibitors, respectively. The mathematical form *ω *was chosen so as to be monotonic and to be bounded in the closed interval [0,1] given that 0≤*x*≤1, *α*>0 and *β*>0. Figure 9 shows the behavior of *ω *when a node is controlled only by one activator. Notice that regardless of the value of *α*, the function is monotonically increasing and bounded to [0,1]. The reason for choosing a monotonic bounded function for *ω *is to preserve the sigmoid form of the total activation acting upon a node *x*_*i*_, irrespective of the number and nature of the regulatory inputs acting upon it. Indeed, Figure 10 shows the total activation of a node *x*_*i *_controlled by one positive regulation with different weights. Notice that the total activation retains a bounded sigmoid form independently of the value of *α*. This same qualitative behavior for total activation on a node *x*_*i *_is observed if it is regulated only by inhibitors. Figure 11 shows *ω *as a function of one inhibitor, plotted for different strengths of interaction. In this case, the total input to *x*_*i *_is still a bounded sigmoid regardless of the value of the parameter *β *(see Figure 12). This general qualitative behavior persists even with a mixture of activatory and inhibitory inputs acting upon a node. Figure 13 presents the total activation of a node *x*_*i *_as a function of two regulatory inputs, one positive and one negative. Notice again that the equation warrants a bounded sigmoid form for the total input to a node.

Once a network is translated to a dynamical system using Equation 3, it is necessary to specify values for all parameters. For a system with *n *nodes and *m *interactions, there are *m*+2*n *parameters. However, there are usually insufficient experimental data to assign realistic values for each and every one of the parameters. Nevertheless, it is possible to use a series of default values for all the parameters in Equation 3. The reason is that, as we showed in the previous paragraph, the equations have the same qualitative shape for any value assigned to the parameters. Hence, for the sake of simplicity, it is possible to assign the same values to most of the parameters, as a first approach. For the present study on the Th model, we use a value of 1 for all *α*s, *β*s and *γ*s; and we use *h *= 10, since we currently lack quantitative data to estimate more realistic values. Moreover, the use of default values ensures the possibility of creating the dynamical system in a fully automated way. Nonetheless, after the initial construction and analysis of the resulting system, the modeler may modify the values of the parameters so as to fine-tune the dynamical behavior of the equations, whenever more experimental quantitative data become available. The continuous dynamical system of the Th model, constructed with the use of Equation 3, yields a system of 23 equations, which is included in the file "Th_continuous_model.octave.txt".

### Stable steady states of the continuous system

Nonlinear systems of ordinary differential equations are studied numerically. Hence the continuous dynamical system defined by Equation 3 poses the problem of how to find all its stable steady states without using very time-consuming and computing-intensive methods. This is where the creation of two dynamical systems of the same network, one discrete and one continuous, bears fruit. Since a Boolean (step) function is a limiting case of a very steep sigmoid curve, networks made of binary elements share many qualitative features with systems modeled using continuous functions [68]. Indeed, it has been shown [19] that the qualitative information resulted from generalized logical analysis can be directly used to find the number, nature and approximate location of the steady states of a system of differential equations representing the same network. We therefore decided to use this characteristic to speed up the process of finding all the stable steady states in the continuous dynamical system. Specifically, the stable steady states of the discrete system are used as initial states to solve the differential equations, running them until the system converges to its own stable steady states. Calculating the convergence of a system of ordinary differential equations from a given initial state is a straightforward procedure using any numerical solver. For our simulations we used the *lsode *function of the GNU Octave package , stopping the numerical integration when all the variables of the system changed by less than 10^-4 ^for at least 10 consecutive steps of the procedure. The final values of the variables in the system are considered to be the stable steady states of the continuous model of the network.

## Implementation

The methodology was fully implemented in a java program, and it has been tested under a linux environment using java version 1.5.0 (JRE 5.0), as well as octave version 2.1.34. The bytecode version of the program is included as Additional file 2.

## Competing interests

The author(s) declare that they have no competing interests.

## Authors' contributions

LM inferred the regulatory network, created the equations, developed the methods and wrote the paper. IX made a substantial contribution to the design and development of the methods, revised the intellectual content, and helped in drafting the manuscript.

## Supplementary Material

Additional File 1 The file contains the set of differential equations describing the continuous version of the Th model. It is a plain text file formatted for running simulations using the GNU Octave package Click here for file

Additional File 2 The file is a java program that implements the methodology described in this paper; it requires a working installation of GNU Octave . The program takes as input a plain text file containing the topology of the network to analyze, with the following format: MoleculeA -> MoleculeB MoleculeB -| MoleculeA The output of the program is a stream of plain text formatted for GNU Octave. Click here for file
